# Zinc protoporphyrin binding to telomerase complexes and inhibition of telomerase activity*

**DOI:** 10.1002/prp2.882

**Published:** 2021-11-08

**Authors:** Zhaowen Zhu, Huy Tran, Meleah M. Mathahs, Brian D. Fink, John A. Albert, Thomas O. Moninger, Jeffery L. Meier, Ming Li, Warren N. Schmidt

**Affiliations:** ^1^ Department of Internal Medicine and Research Service Veterans Affairs Medical Center Iowa City Iowa USA; ^2^ Department of Internal Medicine Roy G. and Lucille A. Carver College of Medicine University of Iowa Iowa City Iowa USA; ^3^ Central Microscopy Research Facility Roy G. and Lucille A. Carver College of Medicine University of Iowa Iowa City Iowa USA

**Keywords:** cancer chemotherapy, telomerase enzymatic activity, telomerase reverse transcriptase, telomere, zinc protoporphyrin

## Abstract

Zinc protoporphyrin (ZnPP), a naturally occurring metalloprotoporphyrin (MPP), is currently under development as a chemotherapeutic agent although its mechanism is unclear. When tested against other MPPs, ZnPP was the most effective DNA synthesis and cellular proliferation inhibitor while promoting apoptosis in telomerase positive but not telomerase negative cells. Concurrently, ZnPP down‐regulated telomerase expression and was the best overall inhibitor of telomerase activity in intact cells and cellular extracts with IC_50_ and EC_50_ values of ca 2.5 and 6 µM, respectively. The natural fluorescence properties of ZnPP enabled direct imaging in cellular fractions using non‐denaturing agarose gel electrophoresis, western blots, and confocal fluorescence microscopy. ZnPP localized to large cellular complexes (>600 kD) that contained telomerase and dysskerin as confirmed with immunocomplex mobility shift, immunoprecipitation, and immunoblot analyses. Confocal fluorescence studies showed that ZnPP co‐localized with telomerase reverse transcriptase (TERT) and telomeres in the nucleus of synchronized S‐phase cells. ZnPP also co‐localized with TERT in the perinuclear regions of log phase cells but did not co‐localize with telomeres on the ends of metaphase chromosomes, a site known to be devoid of telomerase complexes. Overall, these results suggest that ZnPP does not bind to telomeric sequences per se, but alternatively, interacts with other structural components of the telomerase complex to inhibit telomerase activity. In conclusion, ZnPP actively interferes with telomerase activity in neoplastic cells, thus promoting pro‐apoptotic and anti‐proliferative properties. These data support further development of natural or synthetic protoporphyrins for use as chemotherapeutic agents to augment current treatment protocols for neoplastic disease.

## Graphical abstract

ZnPP is a metalloprotoporphyrin that has been investigated as a potential chemotherapeutic agent. ZnPP was found to inhibit telomerase activity, bind to telomerase complexes, and co‐label telomerase and telomeres in situ. These data support further development of natural or synthetic protoporphyrins to augment current treatment protocols for neoplastic disease.

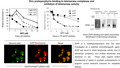

**Abbreviations**
ANOVA: analysis of variance
CoPP: cobalt protoporphyrin
FePP: iron protoporphyrin (heme)
MPP: metalloprotoporphyrin [Fe, Zn, Sn, Co]
MTT: 3‐(4,5‐dimethylthiazol‐2‐yl)‐2,5‐diphenyltetrazolium bromide
NS: nonstructural replicon of HCV
RT‐PCR: reverse transcription ‐ polymerase chain reaction
SnPP: tin protoporphyrin
SYBR: *N*’, *N*'‐dimethyl‐*N*‐[4‐[(E)‐(3‐methyl‐1,3‐benzothiazol‐2‐ylidene)methyl]‐1‐ phenylquinolin‐1‐ium‐2‐yl]‐*N*‐propylpropane‐1,3‐diamine
TERC: telomerase RNA component
TERT: telomerase reverse transcriptase
TMPyP4: tetra‐(N‐methyl‐4‐pyridyl)porphyrin
TRAP: telomerase repeat amplification protocol
WB: western blot
ZnPP: zinc protoporphyrin

## 1 INTRODUCTION

Telomerase is a cellular reverse transcriptase that is reactivated in about 85% of all cancers.^1^ The enzyme maintains adequate lengths of chromosomal 3’ DNA telomeric strand ends, which are continuous sequences of –(TTAGGG)n‐‐ that progressively shorten with each replication cycle because of the DNA polymerase 3’ end replication problem. Using an RNA template, telomerase adds complementary DNA bases to the 3’ telomere end which prevents chromosomal end damage and enables prolonged cellular proliferation, the hallmark of cancer cells. In rapidly dividing malignant cells, telomeres need constant repair to enable high replication rates.^2^ This activity is so crucial to malignancy that even in the 15% of neoplastic cells that do not express telomerase, telomeric ends are maintained by an alternative recombination process.^3^

Considerable evidence supports the feasibility of telomerase inhibitors as chemotherapeutic agents^4^, ^5^, ^6^ for a number of neoplastic diseases. As a class, planar, positively charged polyaromatic compounds such as porphyrins have been shown to have anti‐telomerase activity.^7^ Porphyrins are known to bind and stabilize single‐stranded telomeric DNA sequences at guanine secondary structures known as quadruplexes (G4)^8^ and impact telomerase presumably through substrate inhibition.^7^, ^9^, ^10^

Metalloprotoporphyrins (MPP) such as FePP and ZnPP, represent a subclass of important naturally occurring porphyrins that are also known to bind G4 structures in general^11^ and some telomeric sequences specifically.^12^, ^13^ Additionally, ZnPP and conjugated derivatives such as ZnPP‐polyethylene glycol have been widely studied in experimental rodent systems for use as chemotherapeutic agents.^14^, ^15^ Considering the widespread interest in porphyrins as telomerase inhibitors as well as work with ZnPP as a chemotherapeutic agent, it is surprising that no studies have addressed potential interactions of ZnPP and other MPPs with telomerase.

The aim of the present study was to determine whether common MPPs impact telomerase expression and enzymatic activity in established hepatoma cells. We show that ZnPP abruptly halts DNA synthesis and promotes apoptosis, while concomitantly depressing the expression of telomerase as well as other proliferative proteins such as cyclin D1 and β‐catenin. Furthermore, ZnPP effectively inhibits telomerase activity in intact cells, crude cellular lysates, and immunoprecipitates (IP) while localizing to large protein complexes that contain telomerase. The effects of ZnPP on apoptosis and toxicity were observed primarily in cells that contain telomerase, thus directly implicating a role for telomerase inhibition in the chemotherapeutic activities of ZnPP. These data indicate that ZnPP and perhaps other natural or synthetic MPPs should be studied further for use as chemotherapeutic agents in a wide range of telomerase positive neoplasms.

## 2 MATERIALS AND METHODS

### 2.1 Materials

*Taq* DNA polymerase (*Perkin*‐*Elmer Cetus*, Norwalk, CT), and Moloney murine leukemia virus reverse transcriptase (*Gibco*/*BRL Life Technologies*, Gaithersburg, MD) were used in these studies. Electrophoresis supplies were purchased from *Bio*‐*Rad* (CA).

All MPPs were obtained from *Frontier Scientific*, *Inc* (Logan, UT) and were >97% purity (ZnPP Zn625‐9, SnPP Sn749‐9, CoPP Co654‐9 and FePP H651‐9). For MPP structures, see Figure S1. MPPs were dissolved in minimal volumes of dimethyl sulfoxide (DMSO) and diluted into culture media or assay buffers to achieve the final concentration. Controls received an identical volume of diluted solvent only. BIBR1532 was obtained from *Cayman Chemical* (Ann Arbor, MI. Item No. 16608). Colcemid was purchased from *Roche Diagnostics GmbH* (Mannheim, Germany. Cat. No. 10295892001).

α‐^32^P‐dGTP (6000 Ci/mmol) was obtained from *Perkin*‐*Elmer* (Waltham, MA. #BLU514Z). ^3^H‐thymidine (86 Ci/mM) was from *Amersham* (Little Chalfont, U.K. TRK‐758*)*.

### 2.2 Antibodies and probes

See Table 1.

**TABLE 1:** List of antibodies, sources, and working dilutions

Antibody	Source	Manufacturer #	Dilution
TERT	*Abcam*	Ab32020, Clone Y182	1:1000 WB 1:100 IF
	*EMD Millipore*	MABE14	2 μg/ml IP
Secondary Abs	*Cell Signaling Tech*	7076 7074	1:3000 WB 1:3000 WB
Actin	*Sigma‐Aldrich*	A2066	1:1000 WB
Alexa Fluor 488 antibody	*ThermoFisher Scientific*	A11001 A11008	1:1000 1:1000
GAPDH	*Santa Cruz*	SC 365062	1:2000 WB
β‐Catenin	*Cell Signaling Tech*	L54E2	1:2000
Cyclin‐D1	*BO Pharmingen*	556470	1:1000
Telomere (Telc‐Alexa 488)	*PNA Bio Inc*	F1004	500 nM
Protein‐G Agarose	*ThermoFisher* *Scientific*	20398	
Bulk IgG (rabbit or mouse)	*Santa Cruz*	sc‐2027 sc‐2025	1–10 mg/ml
Cyclin A2	*Cell Signaling Tech*	#4656	1:500 WB
Dyskerin	*Santa Cruz*	sc‐48794	1:1000 WB

Note

Antibodies and immunological probes listed in the text were collated for reader convenience and easy access. Dilutions reflect titers used for assays as described further in the text.

### 2.3 Cell lines and cell culture

Huh7, Hek293, and the HCV permissive clonal line Huh7.5 cells were maintained in routine cultures as described.^16^ The human hepatoma cell line (Huh5.15) with replicating sub‐genomic HCV RNA (genotype 1b) (Huh5.15NS)^17^ was cultivated as described.^18^ Wild type Hek293 cells were obtained from University of Iowa Tissue Culture stocks and passed routinely in minimal essential medium containing 10% fetal bovine serum. Telomerase negative U2OS osteosarcoma cells were obtained from *American Type Culture Collection* and passed using recommended media conditions. U2OS extracts were routinely tested by immunoblot analysis to ensure TERT negativity.

### 2.4 Vectors and constructs

*Lipofectamine^T^* ^M^ 2000 (*ThermoFisher)* was used for all transfections and closely followed the manufacturer's protocol. For TERT overexpression the catalytically active TERT plasmid pCI neo‐hEST2, a gift from Dr. Robert Weinberg (*Addgene* plasmid # 1781)^19^ was used. Telomerase RNA component (TERC) plasmid (pBS U3‐hTR‐500) was also obtained from *Addgene (*plasmid # 28170), a gift from Dr. Kathleen Collins.^20^, ^21^

### 2.5 DNA synthesis, cellular proliferation and apoptosis

#### 2.5.1 DNA synthesis

Semi‐quantitative differences in DNA synthesis were determined by measuring ^3^H‐thymidine uptake into whole cells in log phase growth. At indicated time points, cell cultures were washed with serum‐free, thymidine deficient medium, then incubated with fresh medium containing 1 μCi/ml of ^3^H‐thymidine for two hours at 37°C. Cultures were washed twice with PBS while attached to dishes, then lysed with 0.5 M NaOH and the relative amount of ^3^H‐thymidine quantified by scintillation counting.

#### 2.5.2 Cell proliferation

MPPs were tested for effects on cell proliferation and viability using MTT (3‐(4,5‐dimethylthiazol‐2‐yl)‐2,5‐diphenyltetrazolium bromide) dye conversion assay (*Cell Titer 96*, *Promega*) as we described^22^ with some modifications. Cells were plated into 96 well plates and allowed to attach overnight. MPPs were added to the cultures and then incubated for the times indicated. At assay time, MTT reagent was added and absorbance measured at 570 nm. Controls included buffer blanks containing MPP because of background absorbances reported for MPPs by us^22^ and others.^23^ The formula used for determination of viable cells relative to controls was:

$$\%\;\;\text{viable}\;\text{cells}=\left({\text{abs}}_{\text{sample}}‐{\text{abs}}_{\text{blank}}\right)/\left({\text{abs}}_{\text{conrtrol}}‐{\text{abs}}_{\text{blank}}\right)\times 100$$

Cell viability was also directly determined with trypan blue staining as previously described and closely correlated with MTT assay.^24^

#### 2.5.3 Apoptosis assay

For detection of MPP apoptotic effects, cells were treated with MPPs (0, 2.5, 5 and 10 µM) for 48 h and assayed using PE Annexin V Apoptosis Detection Kit (Cat. No. 559763, *BD Biosciences*) as recommended by the manufacturer.

### 2.6 Quantification of telomerase activity by real‐time quantitative PCR and TRAP assay

Telomerase reverse transcriptase (TERT) enzymatic activity was determined using *Telomeric Repeat Amplification Protocol* (TRAP) with *Real‐time* quantification as described previously,^25^ or measured directly using α‐^32^P‐dGTP incorporation as described^26^ with modifications as below.

For TRAP assay, a Quantitative *Telomerase Detection Kit* (*US Biomax*, Inc) was used to assay cellular lysates or TERT immunoprecipitates (IP) according to the manufacturer's directions. Relative telomerase activity was derived from a standard curve of reference samples and data were analyzed using relative fluorescence units as compared to controls. In some cases, telomerase reaction products were visualized and quantified with *TRAPeze* system (*EMD*/*Millipore*). After separation of the DNA products using 10% non‐denaturing PAGE and SYBR fluorescence labeling [1x SYBR safe DNA gel stain (*Invitrogen)*], gel bands were imaged with *iBright 1500* (*Invitrogen)*. The intensity of the sample's TRAP ladder and internal control was first measured using *GelAnalyzer* [*GelAnalyzer* 19.1 (www.gelanalyzer.com )] as recommended. Then, the relative telomerase activity was determined by the ratio of the intensity of the sample's TRAP ladder (telomerase products, TP) to that of the internal control (IC) band.

### 2.7 Direct telomerase activity assay

For direct telomerase activity assay, a modified procedure of Tomlinson et al^26^ was used. Briefly, HEK‐293T cell pellet from 10^7^ cells overexpressing TERT, TERC, and dyskerin was obtained from *Abbexa Ltd* (UK. Abx069991). The whole‐cell lysate was produced using 1ml buffer A [20 mM HEPES‐KOH buffer (pH 8), 300 mM KCl, 2 mM MgCl_2_, 0.1% v/v Triton X‐100, 10% v/v glycerol]. Immunoprecipitation of telomerase was performed with anti‐hTERT polyclonal sheep antibody (abx120550, *Abbexa*, U.K.). The telomerase was finally eluted with hTERT peptide (abx069990, *Abbexa*, U.K.). 10µl of eluates were mixed with different concentrations of MPP and incubated on ice for 2 h. Then the pellet‐MPP complex was added to the extension reaction mixture composed of 1x telomerase buffer, 300 mM KCl, 1 µM Bio‐L‐18GGG, 1 mM dATP, 1 mM dTTP, 10 µM dGTP, 20 µCi α‐^32^P‐dGTP, 10 mM DTT, 5’ biotinylated DNA substrate (5’‐CTAGACCTGTCATCA(TTAGGG)_3_‐3’) and 10% glycerol. The reaction was conducted at 37ºC for 2 h. The purification of telomerase extension products employed *Dynabeads* M‐280 streptavidin. Five microliters of purified products were loaded on 6% sequencing gel (TBE‐UREA denaturing gel) with Model S2 Sequencing Gel Electrophoresis Apparatus (*LabRepCo*, Horsham, PA). The gel was dried at 80^o^C for 30 min and exposed to phosphorimaging screen for 2 h. This screen was then scanned using a STORM phosphorimager (*Molecular Dynamics*), and bands were quantified using *GelAnalyzer* as described above.

The EC_50_ was the extracellular concentration of MPP inhibitor calculated to result in 50% reduction of cellular telomerase activity after incubation in whole cells. Similarly, IC_50_ was the concentration of MPP inhibitor necessary to inhibit 50% of telomerase enzyme activity in vitro in cellular lysates. Both were calculated using *GraphPad* as directed and verified graphically on plots of enzyme activity vs inhibitor concentration.

### 2.8 Non‐denaturing agarose gel electrophoresis

0.8% Agarose gels were run in Tris‐Borate‐EDTA buffer using standard slab gels as described for high molecular weight complexes.^27^ Cell lysates were produced by lysing Hek293 and Huh7 cells in NP40 buffer (25 mM HEPES‐KOH, 150 mM KCl, 1.5mM MgCl_2_, 10% glycerol, 0.5% NP40, 5 mM 2ME, pH 7.5 supplemented with protease inhibitors) for 30 min on ice. Extracts were clarified by centrifugation for 16 000 *g* for 10 min. The protein concentration was determined by Bradford assay. The indicated amounts of proteins were treated with different amounts of ZnPP for 2 h on ice, and then separated on 0.8% Agarose gels prepared with 0.5xTris‐Borate‐EDTA (TBE) buffer. The gels were run at 100 V for 2 h at 4ºC in 0.5x TBE buffer. To further demonstrate the binding of ZnPP to telomerase complex, immunoprecipitation was performed using 300µg of Hek293 lysate with hTERT antibody (Y182, *Abcam*). No antibody and normal rabbit IgG (sc‐2027, *Santa Cruz Biotechnology*) were included as controls. The IP products were treated with ZnPP and electrophoresed as above. Free ZnPP and ZnPP bound to large complexes were visualized using a wide‐wavelength UV lightbox or with red fluorescence using excitation (EX) 608–632 nm and emission (EM) 675–720 nm. Thyroglobulin (670 kD), a heavy MW marker, was run in parallel lanes and visualized with Coomassie Blue staining or fluorescence to size the complexes. In some cases, cell lysates or IP were incubated with RNase A (*Life Technologies*, NY); or DNase 1 (*Qiagen*, CA) 100 µg/ml for 1 h at 4ºC prior to electrophoresis.

*Diffusion blotting of 0*.*8% agarose native gels*: After electrophoresis, protein complexes were transferred to nitrocellulose (NC) membranes using direct capillary action overnight at RT in the presence of 1x Tris‐*buffered* saline (TBS). Then the NC membranes were washed with fresh TBS and immunodetection of complexes performed as described below for western blot assays.

### 2.9 TERT overexpression and Immunoprecipitation

Plasmid pCl neo‐hEST2 together with TERC (pBS U3‐hTR‐500) were transfected into log phase Hek293 cells and non‐denaturing cellular lysates were prepared 48 h later in lysis buffer (*Cell Signaling Technology*, Beverly, MA). Immunoprecipitation was performed as described previously.^21^ Briefly, transfected cells were harvested, washed in PBS, lysed in cell lysis buffer, and clarified by cold centrifugation (14 000× *g* for 10 min). An aliquot of supernatant containing 500 µg protein was incubated with 2 µg anti‐hTERT antibody MABE14 (*EMD Millipore*, MA) at 4ºC overnight with gentle mixing. Then, 20 µl of recombinant Protein G Agarose (*Invitrogen*, CA) was added and incubated at 4ºC for 3 h. IPs were collected by centrifugation at 3,000 rpm for 30 s at 4ºC, washed three times with ice‐cold PBS, aliquoted, and stored at −80°C until use. For denaturing gel electrophoresis, aliquots were dissolved in 2X Laemmli electrophoresis sample buffer (*Bio*‐*Rad*, CA) and assayed by western blot (WB). Normal rabbit or mouse IgG was always used as a control (*Santa Cruz*, CA). Aliquots of the IP were also assayed in triplicate by TRAP assay and quantified using *realtime* PCR as described above. In some cases, aliquots were electrophoresed on non‐denaturing agarose gels after treatment with MPP and/or nucleases.

### 2.10 *SDS*‐*Polyacrylamide gel electrophoresis (PAGE) and Western blot assays*

For SDS ‐PAGE, cellular lysates, and protein preparations such as IP were dissolved in Laemmli buffer, boiled for 1 min and separated on denaturing SDS gels as described.^28^ After electrophoretic transfer of separated proteins to nitrocellulose sheets, western blot immunoassays employed enhanced chemiluminescence for signal detection (*ECL^TM^ Prime*, *Amersham)*.^28^

### 2.11 Cellular fluorescence labeling

Cells were grown to semi‐confluency while attached to coverslips, then washed in PBS, fixed in absolute methanol, re‐washed in PBS, then incubated with *a*nti‐TERT antibodies with or without 10 μM ZnPP for 1 h at RT. Cells were washed in PBS, then incubated with secondary antibodies conjugated to the fluorochromes *Alexa Fluor 488* (green). Slides were mounted with *VECTASHIELD* H‐1000 (*Vector Labs*, Burlington, Ontario) and counterstained with *To*‐*pro™*‐*3* Iodide (ThermoFisher Scientific) to visualize nuclei. Confocal microscopy was performed on a *Zeiss LSM710* confocal fluorescence microscope. *Alexa Fluor 488* (green) and *Alexa Fluor 568* (red) filters were used to visualize TERT and ZnPP respectively.

### 2.12 Preparation of metaphase chromosomes

Huh7 cells were cultured in regular DMEM with colcemid (0.1 µg/ml) for 2 h at 37°C. Cells were harvested, pelleted by centrifugation, supernatant removed, then re‐suspended in a solution of warm (37°C) 0.075 M KCl, and incubated for 20 min in a 37°C water bath. The cells were pre‐fixed by adding fixative (3:1 ethanol/acetic acid) and centrifuged for 5 min at 1000 RPM at room temperature. The supernatant was removed, and the cells suspended in the fixative solution. The metaphase chromosomes were then spread on slides and reacted with antibodies for immunofluorescence or labeled with ZnPP or telomere probe as described above.

### 2.13 *S*‐*phase synchronized cells*

To synchronize cells in S phase, we performed double thymidine block essentially as described^29^ with modifications. Briefly, Huh7 cells were seeded onto coverslips and then treated with 2 mM thymidine (*Sigma*, T9250) for 18 h, released for 9 h, again treated with 2 mM thymidine for 18 h, then released 2 h before use. To determine the optimal timepoint for collecting cells in S phase, we assayed cultures with flow cytometry and Western blot for cyclin A expression (Figure S4). The cells were fixed with 4% paraformaldehyde and washed with PBS. Telomere FISH and ZnPP staining was then conducted as described below.

### 2.14 Fluorescence in situ hybridization (FISH)

We performed telomere FISH^16^ using a peptide nucleic acid (PNA) probe specific to telomeres, and labeled with TelC‐Alexa488 (*PNA Bio*, Newbury Park, CA,) as per the manufacturer's instructions with modifications. Briefly, 0.2 µl of PNA probe was added to 20 µl of hybridization solution which was used to cover cells attached to slides. Hybridization was at 80°C for 10 min, then 37ºC overnight. After washing, slides were incubated with 10 μM ZnPP 2 h at room temperature, washed twice in PBS, and mounted with *VECTASHIELD*. Confocal fluorescence microscopy used a *Zeiss LSM710* confocal fluorescence microscope using 63x oil objective.

### 2.15 Statistical determinations

All mean values for enzymatic and proliferation assays were determined using 3–6 replicates per point. Data are plotted as the mean value of each point ± standard deviation. For all figures, when a ± bar is not seen, the SD was smaller than the graph symbol. *Graphpad Prism* or *Excel* software was used for least squares regression, calculation of all variances, and curve placement. A completely randomized design with multiple treatment groups was used for the analysis of variance (ANOVA) for each experiment and variances then pooled among experiments using appropriate degrees of freedom for among and within group comparisons. IC_50_ and EC_50_ values (as defined above) were determined by regression assuming sigmoid inhibition curves using *GraphPad Prism* software and tested pairwise using t‐statistic assuming normal distribution. All experiments were repeated at least twice, and the authenticity of the findings verified on three or more occasions.

### 2.16 Nomenclature of targets and ligands

Key protein targets and ligands in this article are hyperlinked to corresponding entries in http://www.guidetopharmacology.org, the common portal for data from the IUPHAR/BPS guide to PHARMACOLOGY^30^ and are permanently archived in the Concise Guide to PHARMACOLOGY 2019/20^31^

## 3 RESULTS

**FIGURE 1:**
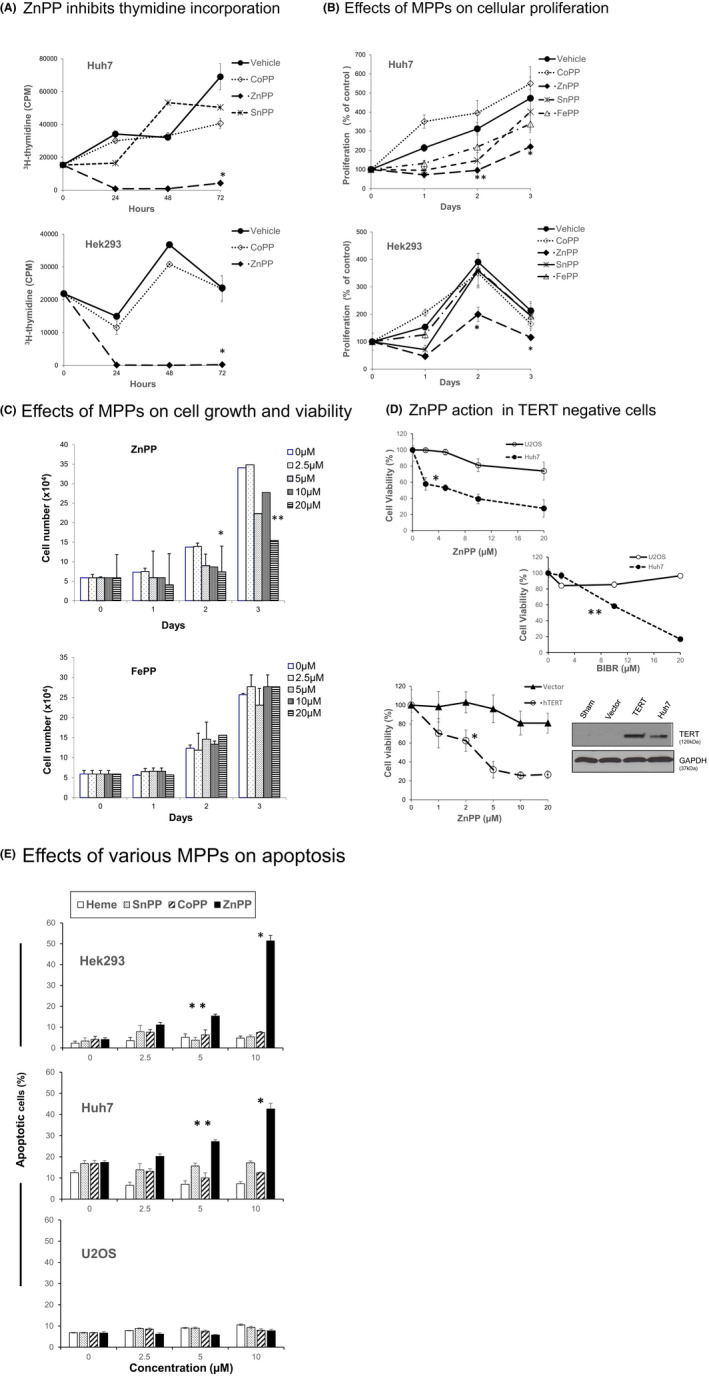
Effects of MPPs on DNA synthesis, cellular proliferation, and viability. (A) Incorporation of ^3^H‐thymidine. Early log phase cultures of Huh7 (upper chart) or Hek293 (lower chart) cells were treated with 10 µM of the indicated MPP. The incorporation of ^3^H‐thymidine was determined at 24, 48, or 72 h of MPP treatment. *t*‐test, ZnPP versus other MPPs,* *p* < .001. (B) Inhibition of proliferation. MTT assays were performed after 10 µM MPP treatment of Huh7 cells (upper chart) or Hek293 cells (lower chart) for 0, 1, 2, 3 days. *t*‐test, ZnPP versus other MPPs, *p < .01, **p < .05. (C) Cell growth and viability. ZnPP (upper chart) and FePP (lower chart) were added to log phase cultures of Huh7 cells. The number of viable cells that excluded trypan blue dye was determined daily using direct cell counting. *t*‐test 5 and 10 µM (*p < .05), and 20 µM **p < .01 versus other concentrations at days 2 and 3, respectively. (D) Effects of ZnPP in TERT negative (U2OS) as compared to TERT positive (Huh7) cell lines. Early log phase cells were treated with the indicated concentrations of ZnPP for 48 h (upper chart) or BIBR for 72 h (middle chart) and viability then determined using MTT assay. Upper chart, *t*‐test U2OS versus Huh7 *p < .01. Middle chart, *t*‐test, U2OS versus Huh7 at 10 and 20 µM *p < .01. For lower chart, vector only plasmid or plasmid pCI neo‐hEST2 containing complete human TERT DNA sequences were transfected into log phase U2OS cells. 24 h later, cultures received the indicated concentrations of ZnPP and then incubated 48 h, followed by MTT assay to determine viability. *t*‐test, TERT vs vector control cells viability *p < .01. (Right) Immunoblot analysis of TERT in U2OS cells after sham treatment or transfections with plasmid only or human TERT sequences. Untreated Huh7 cell lysate with endogenous TERT is shown as positive control. (E) Apoptosis was measured with BD Biosciences Annexin V FITC assay in Hek293 (upper), Huh7 (middle) and U2OS (lower) panels cells after incubation with the indicated concentration of MPP for 48hr. ANOVA with paired t‐ test, ZnPP apoptosis versus other MPPs,**p < .025, *p < .01. (A‐E) Variability expressed as mean, +/‐ standard deviation, *n* = 3–6 determinations per point

We first compared the effects of various MPPs (FePP, ZnPP, SnPP and CoPP) on cellular proliferation and DNA synthesis, (Figure 1A‐C) in Huh7 hepatoma and Hek293 embryonic kidney cells known to express telomerase. In contrast to other MPPs, ZnPP severely attenuated DNA synthesis (Figure 1A) and depressed cellular proliferation greater than 50% at 48 h in both cell lines relative to vehicle only controls (Figure 1B‐C).

Further experiments documented that ZnPP had only minor effects on proliferation in telomerase negative U2OS cells, (Figure 1D, upper chart). As a positive control, BIBR1532, a known mixed‐type non‐competitive inhibitor of telomerase^32^ was tested in the same cell lines. BIBR also showed greater propensity to decrease proliferation in telomerase‐expressing rather than a telomerase negative line in accordance with earlier reports^33^ (Figure 1D, middle chart). In contrast, transfection of TERT sequences into U2OS restored ZnPP sensitivity that was quite similar to that of Huh7 cells (Figure 1D, upper and middle charts respectively). The immunoblot of Figure 1D (lower panel) documented that TERT transfected U2OS cells avidly expressed TERT as compared to vector‐only controls.

Consistent with effects on DNA synthesis and proliferation, ZnPP was the most effective MPP at inducing apoptosis (ca.: 50% at 10 µM) in TERT positive Huh7 or Hek293 cells in contrast to other MPPs evaluated (Figure 1E, upper and middle charts respectively). On the other hand, ZnPP failed to have an increased effect on apoptosis in telomerase negative U2OS cells (Figure 1E lower chart). Taken together, these findings suggest that ZnPP interactions with the telomerase system contribute to the effects on apoptosis and anti‐proliferative behavior.

**FIGURE 2:**
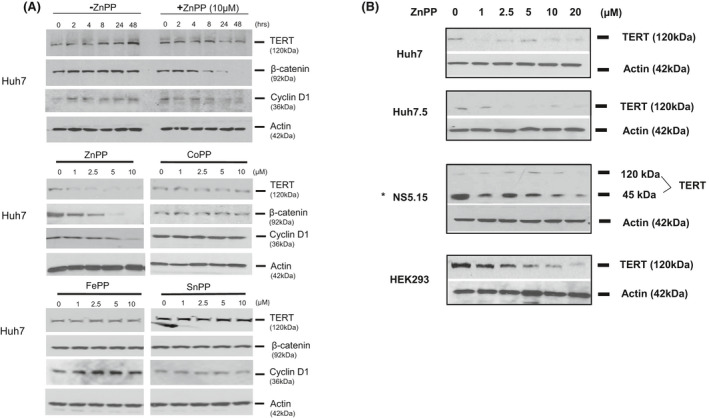
MPP effects on cellular expression of proliferative proteins TERT, β‐catenin, and cyclin D1. (A) ZnPP decreases expression of proliferative proteins. Log phase Huh7 cells were treated with the indicated concentrations of MPPs and assayed for various times, (upper panel) or after 48 h (middle and lower panels) on WB using specific antibodies. (B) Log phase cultures of different clonal Huh7 and Hek293 cell lines were incubated with the indicated concentrations of ZnPP for 48 h. TERT was then identified on WB with specific anti‐TERT antibodies. [*HCV positive replicon ^21^]

Because of ZnPP actions on DNA synthesis, proliferation, and apoptosis in TERT positive cells, we determined ZnPP effects on TERT expression with western blots (WB) (Figure 2A). As positive controls, we also evaluated other pro‐proliferative proteins, β‐catenin and cyclin D1 which have been closely linked to TERT expression and signaling.^34^, ^35^ ZnPP reduced expression of all three proteins by 8−24 h and by 48 h expression was nearly eliminated (Figure 2A). Cells treated with various concentrations of ZnPP for 48 h (Figure 2A, left middle panel) further confirmed these findings. In contrast, CoPP, FePP, or SnPP failed to significantly alter TERT, β‐catenin, or cyclin D1 expression (Figure 2A, right middle and bottom panels) consistent with their minimal effects on DNA synthesis and apoptosis (Figure 1). The effects of ZnPP on TERT expression were apparent in different Huh 7 constructs and, interestingly, in a NS 5.15 HCV replicon, ZnPP promoted disappearance of both 120 kDa telomerase monomer as well as the 45 kDa C‐terminal TERT fragment that we previously reported to be specific for HCV infected cells (Figure 2B).

^21^

**FIGURE 3:**
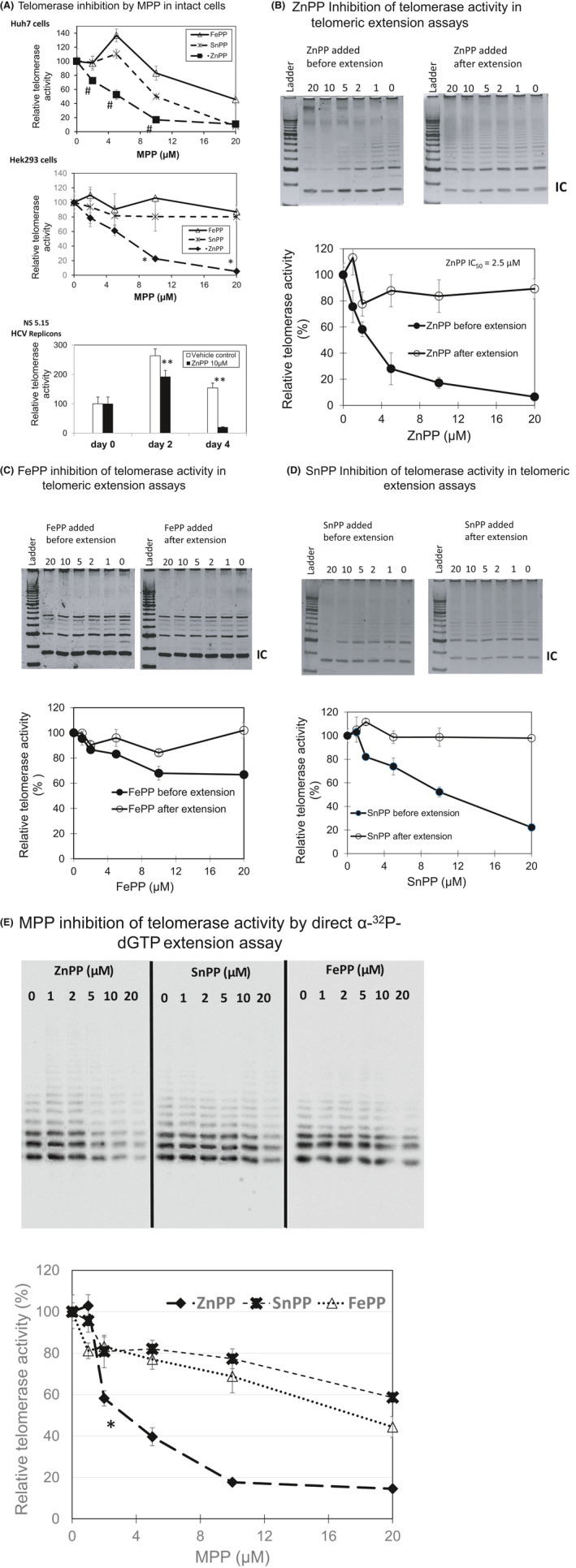
MPP inhibition of telomerase enzymatic activity. (A) Telomerase inhibition by MPP in cultured cells. Log phase Huh7, Hek293 and Huh5.15 HCV replicon cells, (upper, middle, and lower panels, respectively), were incubated with MPPs (ZnPP, SnPP, or FePP) for 48 h, then telomerase activity was determined by TRAP assay in enzymatic lysate. Points represent mean ± SD, *n* = 6, ANOVA, #*p* < .01, *p < .001, **p < .01 paired t‐test of ZnPP versus other MPPs or vehicle only controls. (B‐D) Telomerase inhibition by MPP in enzymatic extracts. Enzymatic extracts were prepared from semi‐confluent phase Huh7 cells and aliquots were assayed in triplicate using TRAP assay. MPPs were added to RT‐PCR reactions either before or after the RT telomere elongation step to test whether MPPs inhibit *Taq* polymerase. Visualization of amplified telomeric products was on 10% non‐denaturing polyacrylamide gels using SYBR fluorescence labeling (upper panels) followed by quantification with densitometry and plotted (lower panel). IC = Internal Taq polymerase control. Plotted points represent the mean ± SD with *n* = 3. (E) MPP inhibition of telomerase activity as determined with direct α‐^32^P‐dGTP extension assays. Aliquots of IP eluates from 10^7^ Hek293 cell pellet lysates overexpressing hTERT, TERC, and dyskerin were incorporated into direct telomere extension assays using biotin‐linked DNA substrate. Reactions were incubated at 37ºC for two hours, then biotin labeled products purified with strepavidin linked agarose beads and electrophoresed on denaturing acrylamide gels. Bands were visualized radiographically (upper panel) and relative activity was quantified with densitometry (lower panel). Points represent mean ± SD, *n* = 3, ANOVA *p < .01 paired t test of ZnPP versus other MPPs

We next evaluated the effects of MPPs on telomerase activity in cultured cells (Figure 3A) as well as non‐denatured cell lysates (Figure 3B‐E). In cultured cells incubated with ZnPP, telomerase activity was reduced in a dose‐dependent fashion, (EC_50_ = 5.6–5.8 µM, upper and middle panels respectively) while SnPP or FePP, had none to mild effects (EC_50_ > 10 µM, either cell line) (Figure 3A). The loss of telomerase activity with time of ZnPP treatment in the NS 5.15 HCV replicon (Figure 3A, lower panel) reflected the disappearance of TERT seen in the WB (Figure 2B).

The possibility that MPPs can directly inhibit telomerase activity in cellular extracts, similar to porphyrin quadruplex ligands,^6^ was addressed next. Because of concerns that some quadruplex ligands inhibit *Taq* DNA polymerase in addition to telomerase, we assayed MPP inhibition at both steps of the TRAP procedure with a strategy similar to that of others.^36^ Using equivalent extracts but separate assays, MPP was either included in the telomerase RT extension step or the extension step was conducted without MPP and then MPP added only for the amplification steps with *Taq* DNA polymerase. To avoid further potential errors introduced by *Real‐time* quantification, TRAP products were labeled with SYBR green, visualized on denaturing gels, and each lane was quantified by density measurements as described in the Methods. The latter step also ruled out the possibility that decreases in activity were artifactual due to fluorescence signal quenching by some MPPs.^37^ ZnPP was significantly more active (IC_50_ = 2.5 µM) than FePP and SnPP (both IC_50_ > 10.0 μM), (Figure 3B‐D respectively). All three MPPs had minimal effects on Taq polymerase during telomerase product extension and the slight inhibition of Taq polymerase seen for ZnPP was not directly concentration‐dependent. However, at least one MPP, CoPP, clearly inhibited Taq polymerase and could not be reliably assayed via TRAP assay (see Figure S2).

To confirm that ZnPP specifically inhibited telomerase as seen in the TRAP assays, direct telomere extension assays were conducted in the presence of α‐^32^P‐dGTP. An IC_50_ of 3.8 μM for ZnPP obtained by direct extension assay (Figure 3E) was quite similar to the IC_50_ obtained with TRAP assay (2.5 μM) (Figure 3B). Consequently, by two independent assay procedures, ZnPP was observed to directly inhibit telomerase activity in cellular extracts and the IC_50_ values are roughly within a twofold range of the EC_50_ values for intact Huh7 and Hek293 cells (5 and 6 μM respectively). IC_50_ and EC_50_ values of the three MPPs obtained by different assay procedures are summarized in Table 2.

**TABLE 2:** Inhibition of telomerase by MPPs in direct telomere extension assays

MPP	IC_50_ (μM)	ME	R^2^	P values of IC_50_ [ZnPP < SnPP or FePP]
a. Cellular lysates [Trapeze]				
ZnPP	2.5	4.39	0.974	
SnPP	12	2.35	0.962	*p* < .001
FePP	>20	7.9	0.911	*p* < .001
b. α−^32^P‐dGTP direct telomerase assay				
ZnPP	3.8	1.64	0.904	
SnPP	>20	2.32	0.645	*p* < .01
FePP	18.3	2.75	0.777	*p* < .01
c. Intact Huh7 cells [TRAP]				
	**EC_50_ (µM**)			
ZnPP	5.4	2.89	0.941	
SnPP	10.8	4.76	0.865	*p* < .01
FePP	>20	9.6	0.405	*p* < .01
d. Intact Hek293 cells [TRAP]				
ZnPP	6.4	2.41	0.928	
SnPP	>20	NC	0.076	*p* < .001
FePP	>20	NC	0.085	*p* < .001
e. TERT IP vs cellular lysate [TRAP]				
	**IC_50_ (µM)**			**IC_50_ of IP vs lysate**
ZnPP (IP)	2.4	4.8	0.953	NS
ZnPP (lysate)	1.8		0.963	

Note

ZnPP, SnPP and FePP telomerase inhibition in intact cells, cellular lysates and anti‐TERT immunoprecipitates. R^2^ values reflect regression analysis assuming sigmoidal inhibition curve. IC _50_ and EC_50_ values were determined as directed by *GraphPad* software. IC_50_ and EC_50_ values are presented as +/‐ the margin of error (ME) determined at 99% confidence level or greater assuming t distribution. Each IC_50_ or EC_50_ reflects mean values of 2–4 experiments using 6 concentrations of inhibitor per curve (0, 1, 2.5, 5, 10, and 20 µM) and 3–6 determinations per point. Mean values may differ slightly from values reported in the text for individual experiments.

ZnPP, SnPP, and non‐metal, “free” Lewis base protoporphyrins exhibit autofluorescence^37^; a property that has proven useful to study intracellular activities of MPPs such as nuclear localization and DNA or cellular adduct binding.^13^, ^23^, ^38^, ^39^ Other transition metal MPPs such as FePP or CoPP are inactive fluoroscopically because they have unfilled transition metal *d* orbitals that quench fluorescence emission. We investigated ZnPP binding to native, non‐denatured telomerase‐containing complexes after separation on large pore, (0.8%), agarose gels.^27^, ^40^ Initially, non‐denaturing acrylamide gels were considered for these studies, however, we discovered that ZnPP labeled complexes would not enter the largest pore size possible, a result also noted by others^23^, ^39^ (see Figure S3).

**FIGURE 4:**
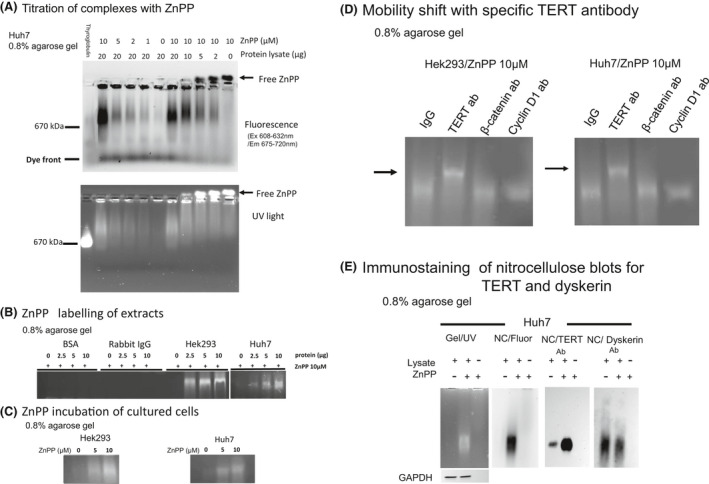
ZnPP fluorescent labeling of cellular extracts. (A) Titration of complexes with ZnPP. Cellular lysates as described in Methods were prepared from log phase Huh7 cells and incubated 1 h at RT with various concentrations of ZnPP. Mixtures were electrophoresed on non‐denaturing 0.8% agarose gels and visualized fluorescently using red excitation 608–632 nm and emission 675–720 nm (upper panel) or broad band UV light (lower panel). (B) ZnPP labeling of extracts. ZnPP (10 µM) was added to BSA, non‐specific rabbit IgG, or cellular lysates from Hek293 and Huh7 at the indicated concentrations and incubated for 1 h at RT. Aliquots were then electrophoresed on 0.8% agarose gels and visualized under broad band UV light. (C) Labeling complexes with ZnPP in intact cells. ZnPP (0, 5, and 10 µM) was incubated with Hek293 and Huh7 cultured cells for 24 h, then enzymatic extracts were prepared, electrophoresed on 0.8% agarose gels, and visualized under broad band UV light. (D) ZnPP labeled complexes have mobility shift with anti‐TERT antibodies. Enzymatic cellular extracts (10 µg as protein) were prepared and labeled with 10 µM ZnPP for 1 h at RT. The indicated antibodies were then added, and mixtures incubated at 4°C for 1 h prior to electrophoresis on 0.8% agarose gels and broad band UV light visualization. (ab = antibody). Arrow = mobility shift of anti‐TERT antibodies. (E) Immunostaining of ZnPP labeled complexes blotted to nitrocellulose. Lysates from Huh7 cells were incubated for 1 h at RT with 10 µM ZnPP or vehicle control, then electrophoresed on agarose gels and diffusion‐blotted onto nitrocellulose (NC). ZnPP complexes were visualized in gels by broad band UV and on NC blots by red fluorescence (wavelengths noted above). NC blots were then stained with specific anti‐TERT or anti‐dyskerin antibodies

Initially, increasing amounts of cellular extracts were incubated with varying amounts of ZnPP, then electrophoresed on large pore agarose gels. ZnPP was then visualized fluoroscopically using visible red wavelengths [608–632 nm Ex and 675–720 nm EM] or wide band UV (Figure 4A upper and lower panels respectively). ZnPP bound to high molecular weight complexes in a concentration dependent manner and the complexes electrophoresed with a mobility just above thyroglobulin (670 kD), quite similar to sizes noted by us and others for TERT ribonuclear protein particles separated by glycerol gradient centrifugation^21^, ^41^ and large pore agarose/acrylamide gels.^40^ Under these conditions, free ZnPP migrated slightly cathodal. While ZnPP binding was easily identified in cellular extracts, no binding was detectable in bulk protein incubations of BSA or IgG (Figure 4B). ZnPP also labeled complexes in intact cells as determined by electrophoresis of extracts prepared after ZnPP incubation in culture (Figure 4C).

To assess whether TERT is a component of the ZnPP‐labeled cellular extracts we incubated lysates with anti‐human TERT, β‐catenin, or cyclin D1 antibodies or non‐specific IgG antibodies prior to electrophoresis and looked for upward mobility shift after electrophoresis (Figure 4D). Only anti‐TERT antibody led to a significant upward mobility shift of ZnPP labeled complexes from either cell type, suggesting that TERT is indeed a component of the large complexes. No mobility shift was noted for the other antibodies tested suggesting ZnPP specifically labeled TERT complexes. Note that both cyclin D1 and β‐catenin would be expected to be components of large molecular complexes in non‐denatured cellular lysates.^42^, ^43^ Further characterization of the ZnPP binding complexes as to protein and DNA composition, and investigation of SnPP binding is presented in the online Supplemental Data (Figure S3).

ZnPP labeled complexes from Huh7 cells were next blotted onto nitrocellulose by capillary diffusion (conditions determined empirically, see Figure S4) and probed with specific anti‐TERT or anti‐dyskerin antibodies, the latter a positive control for telomerase holoenzyme (Figure 4E). Both TERT and dyskerin were easily identified in the high molecular weight complexes binding ZnPP (Figure 4E). Interestingly, cellular lysates showed more immunoreactive TERT when incubated with ZnPP prior to electrophoresis suggesting a protective effect of ZnPP on TERT in the extracts.

**FIGURE 5:**
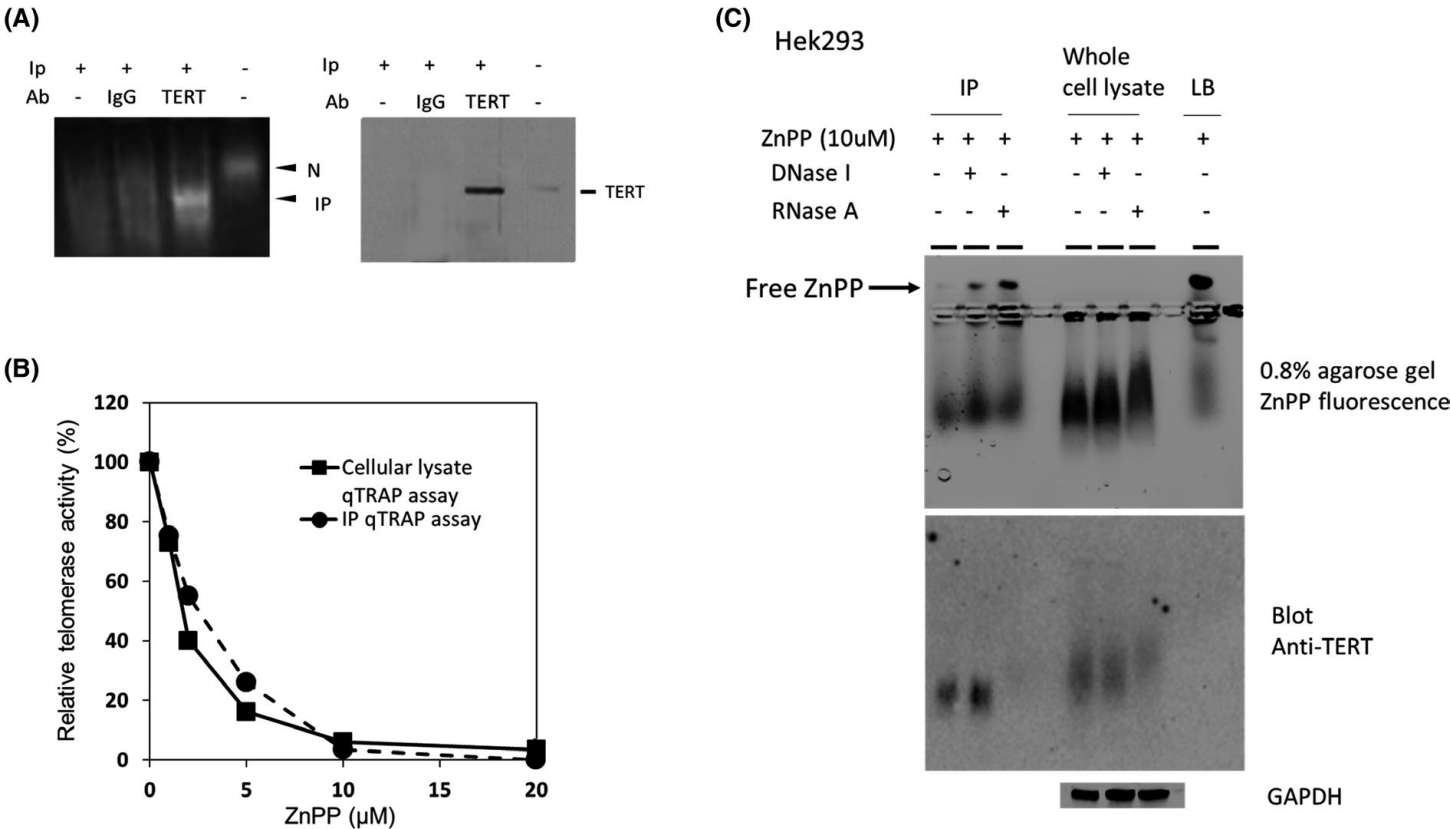
ZnPP inhibition and labeling of Immunoprecipitated TERT complexes. (A) ZnPP binds TERT IP complexes. *Left panel*: Immunoprecipitation was performed on lysates from Hek293 cells transfected with TERT and TERC plasmid constructs as described in the Methods. The IP were then re‐suspended and incubated with ZnPP (10 µM) for 1 h at RT prior to electrophoresis on 0.8% agarose gels and visualized under UV light. *Right panel*: Identical aliquots were also denatured for SDS‐PAGE and evaluated on WB. (B) ZnPP inhibition of telomerase activity in cellular lysates and IP. TRAP assays were performed with IP or crude cellular lysates in the presence of various concentrations of ZnPP. No statistical difference of ZnPP IC_50_ of telomerase from either source (Table 2). (C) Nuclease digestion of ZnPP labeled complexes. *Upper panel*: Enzymatic extracts or IP (10 µg protein) were treated with DNase 1 or RNase A (0.1 mg/ml at 4^o^C for 1 h), then labeled with ZnPP (10 µM) for 1 h at RT, before electrophoresis on 0.8% agarose gels and visualization of ZnPP by red fluorescence emission. *Lower panel*: Following electrophoresis, native proteins were transferred to NC by diffusion blotting and stained with anti‐TERT antibodies. For whole cell lysates, GAPDH was used as a loading control after lysates were electrophoresed on SDS‐denaturing polyacrylamide gels and stained using WB methods (LB = lysis buffer); (IP= immunoprecipitate); (N = Native)

Immunoprecipitation using TERT‐specific antibodies further confirmed that TERT is a component of ZnPP labeled complexes. Immunoprecipitates (IP) were evaluated on native agarose as well as denaturing SDS gels and WB (Figure 5A, left and right panels respectively). ZnPP bound the anti‐TERT IP complexes intensely and IP had increased mobility as compared to non‐specific IP complexes or no antibody control (Figure 5A, left panel). As expected, the anti‐TERT IP analyzed on WB (Figure 5A right panel) showed increased TERT as compared to IP from non‐specific or no antibody controls. A weaker TERT band (relative to exposure time) was also confirmed in the crude cellular lysate. When assayed by TRAP assay, IP TERT complexes and unpurified enzyme showed similar IC_50_ values with ZnPP, (3.1 vs. 2.2 µM, respectively), (Figure 5B). These measurements were also close to the IC_50_ observed for overexpressed enzyme, (3.8 µM), when assayed by direct α‐P^32^‐dGTP extension assay (Figure 3E), (Table 2).

To investigate whether nucleic acids are components of the ZnPP labeled complexes, IP or crude lysates, were digested with DNase I or RNase A prior to labeling with ZnPP (Figure 5C, upper panel). Digestion of extracts with DNase I elicited minimal changes in the mobility of ZnPP labeled complexes, however, a significant upward shift was seen when extracts were digested with RNase A. Furthermore, there was a marked increase in unbound ZnPP after nuclease, most dramatic with RNase A—treated IPs (arrow, Figure 5C, upper panel) suggesting that ZnPP most likely binds to a ribonuclear protein complex and the binding site is at least partially disrupted with RNase digestion. Next, nuclease digested, ZnPP labeled complexes were blotted onto nitrocellulose and reacted with anti‐TERT antibodies. These experiments showed that RNase A digestion severely diminished the amount of immunoreactive TERT in the labeled complexes, most notably for IP complexes (Figure 5C, lower panel). These findings strengthened the conclusion that ZnPP binds to high molecular weight telomerase complexes. In addition to providing a telomere template, TERC is an important structural component of telomerase holoenzyme and RNase A digestion is known to completely disrupt ribonuclear complex structure and release TERT.^44^

**FIGURE 6:**
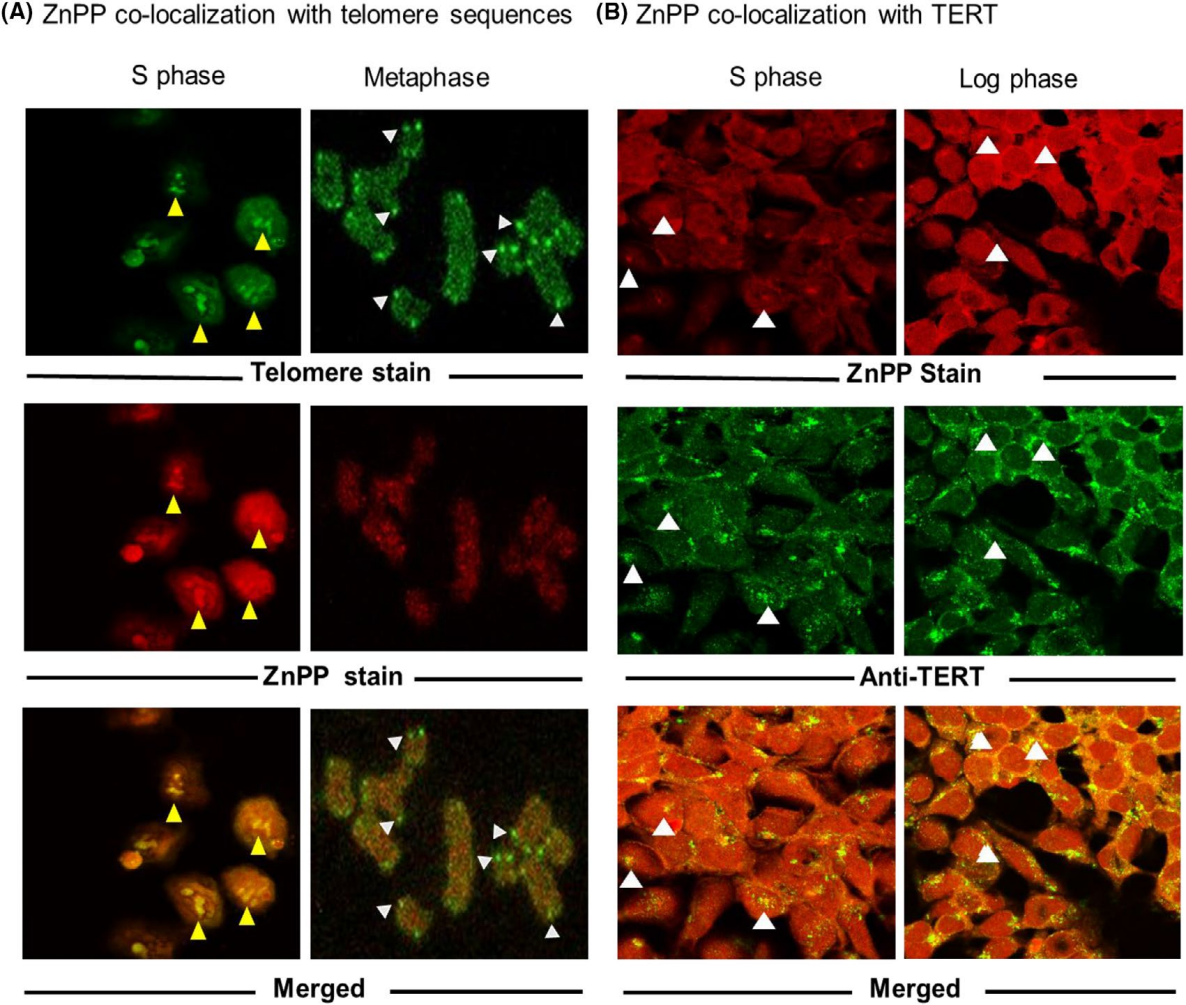
Fluorescence co‐localization of ZnPP with telomeres and TERT. (A) Co‐localization of ZnPP and telomeres. Synchronized S phase Huh7 cells (left panels) and metaphase chromosomal spreads (right panels) were prepared as described in Methods. Fixed preparations were reacted with the specific fluorescent telomere probe TelC‐Alexa488 and 10 µM ZnPP. Confocal microscopy used *Alexa Fluor 488* (green filter) or *Alexa Fluor 568* (red filter) to visualize telomeres or ZnPP, respectively, and merged sites (yellow). Yellow arrows (left panels) show co‐localization of telomeres and ZnPP. White arrows, (*right panels)* show localization of telomeres which do not co‐localize with ZnPP. (B) Co‐localization of ZnPP and TERT in synchronized S phase (left panels) and unsynchronized log phase (right panels). Huh7 cells were incubated with 10 µM ZnPP after labeling with anti‐TERT antibodies. Confocal microscopy was then conducted using *Alexa Fluor 488* (green filter, anti‐TERT) or *Alexa Fluor 568 (red filter*, *ZnPP)* fluorochromes to visualize TERT or ZnPP, respectively, and merged sites (yellow). Arrows depict example sites of co‐localization of ZnPP and TERT in the nuclei of S phase cells (left panels) or cytoplasm/perinuclear regions of unsynchronized semi‐confluent cells (right panels)

ZnPP binding to cellular structures in situ was probed by confocal immunofluorescence microscopy. While telomere sequences exist throughout the mammalian chromosome, it is known that telomerase holoenzyme only associates with telomeres at DNA replication during S phase.^45^ Consequently, we compared ZnPP localization in synchronized S‐phase cells as compared to metaphase chromosomes which contain prominent telomere ends without telomerase. The percentages of cells in S phase were determined temporally with flow cytometry after double thymidine block and extracts were monitored on immunoblots with Cyclin A2 staining to determine optimal times for study, (Figure S5). Telomere sites were labeled with telomere sequence‐specific fluorescent probe (PNA TeIC‐Alexa488) and TERT was localized with specific antibodies (Figure 6).

First, we looked at ZnPP co‐localization with the telomere probe. While ZnPP clearly localized with telomeres in S phase cells (Figure 6A left panels), it did not label the prominent telomeres on metaphase chromosome tips, (Figure 6A, right panels) which are devoid of holoenzyme. Next, we investigated whether ZnPP would co‐localize with TERT in S phase as compared to unsynchronized Huh7 cells. In S phase cells, TERT co‐localized with ZnPP in the nucleus and at some cytoplasmic sites (Figure 6B left panels). As we reported previously, TERT is sparsely present in the nucleus of unsynchronized, log phase Huh7 cells, but is chiefly found at perinuclear sites which co‐localize with mitochondria.^21^ Interestingly, even perinuclear TERT, likely lacking telomeric DNA, showed avid TERT‐ ZnPP co‐localization (Figure 6B, right panels). Collectively, these data indicate that ZnPP can bind to telomerase complexes and/or associated components. While telomeric DNA does not appear to be a primary binding site of ZnPP per se, at least at prominent telomeres on metaphase chromosomes, the specific sites of interaction in the telomerase holoenzyme remain to be determined.

## 4 DISCUSSION

The development of telomerase inhibitors for use in chemotherapy began shortly after the discovery of telomerase over three decades ago. Telomerase inhibitors are cytotoxic to most tumor cells and promote telomere shortening and instability, DNA damage responses, DNA synthesis arrest, apoptosis, and other cellular senescence programs.^6^ Multiple telomerase inhibitors are in development and include competitive, non‐competitive allosteric, and apparent DNA substrate inhibitors [see^5^ for a recent review].

A wide variety of planar aromatic macromolecules and porphyrins have also been shown to inhibit telomerase.^46^ These compounds are thought to stabilize hydrogen‐bonded guanidine‐tetrads (G4 complexes) that form in telomeric DNA and inhibit cyclic realignment of the DNA substrate with TERT during the enzyme cycle, thus reducing telomerase processivity.^9^, ^47^ Functionally, synthetic porphyrins such as tetra‐(N‐methyl‐4‐pyridylporphyrin), (TMPyP4), were shown to cause cell growth arrest and apoptosis in neoplastic cells which demonstrated that G4 telomeric binding sites are useful targets for chemotherapy.^10^, ^48^

In contrast to porphyrins, protoporphyrins have not been investigated for anti‐telomerase behavior. Nevertheless, common MPPs such as FePP and ZnPP are known to bind selected oligomeric G‐4 sequences in vitro,^13^, ^49^ thus suggesting that they are capable of behaving like porphyrins for telomerase inhibition. Biologically, ZnPP and long‐acting conjugates such as ZnPP‐polyethylene glycol have been studied as chemotherapeutic agents in vivo in rodent models for some time^14^ and promote tumor regression, both alone as well as synergistically with drugs such as cisplatin.^15^ Consequently, investigation of ZnPP effects and sites of action on telomerase activity is timely and important.

We first evaluated the effects of common MPPs on telomerase activity, cellular proliferation, DNA synthesis, and apoptosis. ZnPP was the most potent MPP tested and down regulated TERT expression, arrested DNA synthesis, and promoted apoptosis in Huh7 hepatoma cells. In contrast, ZnPP was only minimally active in U2OS cells which contain no telomerase,^50^ however, sensitivity could be acquired following TERT transfection. This suggests that telomerase interactions likely play a role in ZnPP anti‐proliferative and apoptotic behavior. Downregulation of TERT was also accompanied by a reduction in cyclin D1 and β‐catenin which are not only important markers of cellular proliferation and apoptosis per se,^51^ but they have known positive signaling relationships with telomerase.^34^, ^35^ The data shown in Figure 1 also support earlier work that documented the specific anti‐proliferative actions of ZnPP.^23^

ZnPP directly inhibited telomerase enzymatic activity in cellular lysates, IP, and intact cells in culture. This was verified by both TRAP and direct α‐^32^P‐dGTP extension assays with similar IC_50_ (Table 1). To date, this is the first demonstration that ZnPP has direct anti‐telomerase activity and these findings support consideration of ZnPP or related MPP compounds for use in chemotherapeutic programs. These studies also lay groundwork for modeling and development of improved, anti‐telomerase derivatives using a ZnPP‐based model design.

ZnPP is a naturally occurring MPP and it inhibited telomerase at EC_50_ and IC_50_ concentrations of ca 5 μM or less, close to levels that can be achieved physiologically. During normal heme synthesis in reticulocytes, ZnPP is produced at low levels (0.5 μM), however, in times of iron deficiency it can rise to values of 5 μM or higher,^52^ well within the effective anti‐telomerase concentrations seen here. The IC_50_ of ZnPP (Table 2) is also close to the micromolar ranges of IC_50_ reported for other synthetic experimental porphyrins.^6^, ^46^

The major components of the telomerase holoenzyme complex include TERT, dyskerin, p23, Hsp90, TERC and telomerase‐associated protein.^53^ TERT is absent from telomeres until it is assembled into telomerase holoenzyme and then recruited to selective chromosomal telomeric sites at the start of S phase.^45^, ^54^ Telomere addition is known to be coupled to DNA synthesis and the processes likely occur sequentially in neoplastic cells.^45^ While the latter interaction may very well account for the profound depression of DNA synthesis in neoplastic cells, we cannot rule out the possibility that ZnPP also has a direct effect on DNA polymerases.

With the structural and functional characteristics of telomerase in mind, we investigated whether ZnPP can bind at or near holoenzyme complexes. On non‐denaturing agarose gels, ZnPP specifically bound to high molecular weight complexes [>670 kD] in cellular extracts. The binding of anti‐TERT antibodies to ZnPP‐labeled complexes resulted in an upward mobility shift, thus demonstrating that the complexes contained TERT. Both TERT and dyskerin were identified in ZnPP labeled complexes by native agarose gel immunoblot analysis. Consequently, ZnPP can bind native high molecular weight complexes that contain components of the telomerase holoenzyme.

As might be expected, IP showed faster mobility than complexes from crude lysates indicating a smaller, immunopurified preparation. DNase 1 (mostly selective for DS DNA) had minimal effects on ZnPP binding while RNase A digestion led to decreased complex mobility, decreased ZnPP binding, and increased free ZnPP. Moreover, RNase A digestion caused loss of TERT from the complexes, a result that might be anticipated since TERC binds TERT in the ribonuclear protein complex and RNase A treatment disrupts holoenzyme structure.^41^ Collectively, these data demonstrate that ZnPP binds ribonuclear protein complexes containing TERT, however, identification of the definitive binding sites requires further study.

ZnPP has been reported to bind some selected G‐4 structures as well as oligomeric telomere sequences in vitro,^13^ thus, considerable heterogeneity of ZnPP binding should be expected. G‐rich DNA sequences likely modulate DNA structure‐regulatory activities at sites such as gene promoters for enzyme systems in addition to telomerase.^55^ Consequently, we cannot yet account for how other ZnPP targeted G4 sites may contribute to our results here.

Confocal immunohistochemical experiments demonstrated that ZnPP binds at or near telomere/telomerase complexes in S phase cells, which is the only time in the cell cycle when telomerase is found at telomeres.^45^ Surprisingly, telomere rich sites on metaphase chromosomal ends did not overtly bind ZnPP (Figure 6A). Consequently, ZnPP interactions with telomerase complexes appear more complex than just telomere G4 binding and may depend on S phase chromatin structure, site accessibility, and composition of the complexes. Furthermore, these data suggest there may be a primary interaction of ZnPP with TERT or closely associated proteins since there was co‐localization of TERT and ZnPP in cytoplasmic as well as nuclear locations (Figure 6B). Interestingly, earlier work showed that ZnPP and FePP could directly bind other reverse transcriptases such as HIV^56^ also supporting a direct interaction of ZnPP and TERT. More studies characterizing the site(s) of ZnPP binding and kinetics of telomerase inhibition are necessary. Overall, the ability of ZnPP to avidly bind telomere sites in S‐phased cells *in situ* strongly suggests it can impact telomerase activity where and when telomerase is active intracellularly as supported by the kinetic and immunoblot experiments.

Aside from telomerase, other cellular sites, both nuclear and cytoplasmic, have been proposed to be responsible for the pro‐apoptotic qualities of ZnPP. Since ZnPP is both a transcriptional inducer for heme oxygenase‐1 (HO‐1)^57^ and a competitive HO‐1 inhibitor,^58^ earlier studies attributed many ZnPP mechanisms to HO‐1 antagonism. Presently it is not known whether ZnPP actions on the telomerase system are impacted by HO‐1 antagonism and this concept requires further study. ZnPP also inhibited transcriptional promoter sites for cyclin D1^23^ and attenuated Wnt/β‐catenin expression leading to increased apoptosis.^59^ Our proliferation studies support these findings, yet it is not yet clear whether telomerase inhibition may impact these other pathways.

## 5 CONCLUSIONS

In summary, our data indicate that ZnPP interacts with the telomerase enzyme system at two major regulatory points: (1) downregulation of TERT and (2) direct inhibition of telomerase enzymatic activity. Concomitantly, ZnPP attenuates DNA synthesis and cellular proliferation while promoting apoptosis. Structurally, ZnPP binds to TERT containing ribonuclear protein complexes and co‐localizes with a subset of nuclear telomeres that likely contain holoenzyme complexes. Our findings support the use of ZnPP and potentially the development of other synthetic or natural protoporphyrins for use as chemotherapeutic agents in the treatment of neoplastic disease. Ongoing further experiments aim to characterize the binding and inhibition sites of ZnPP in the telomerase complexes.

## 6 ETHICS STATEMENT

This paper is the original and authentic work of the authors. All authors read and approved the final manuscript.

## CONFLICT OF INTEREST

The authors declare there are no conflicts of interests.

## AUTHORS’ CONTRIBUTIONS

Zhu, Tran, Meier, and Schmidt participated in research design. Zhu, Tran, Mathahs, Albert, and Fink conducted experiments. Zhu, Tran, Moninger, Meier, Li, and Schmidt contributed new reagents or analytic tools. Zhu, Fink, Albert, Meier, and Schmidt performed data analysis. Zhu, Tran, and Schmidt wrote or contributed to the writing of the manuscript.

## Supporting information

Fig S1‐S5

Supplementary Material

## Data Availability

All data underlying the results are freely available as part of the article and no additional source data are required.
