# Supplementary material for: Zinc protoporphyrin binding to telomerase complexes and inhibition of telomerase activity
Source: Pharmacol Res Perspect. 2021 Nov 8;9(6):e00882. doi: 10.1002/prp2.882 (PMC8573827; doi:10.1002/prp2.882)
Supplement: Supplementary file 2 — Supplementary Material [file PRP2-9-e00882-s001.docx]

**Zinc protoporphyrin binding to telomerase complexes and inhibition of telomerase activity**

**Supplementary Material**

Zhaowen Zhu, Huy Tran, Meleah M. Mathahs, Brian D. Fink, John A. Albert, Thomas O. Moninger, Jeffery L. Meier, Ming Li and Warren N. Schmidt

Department of Internal Medicine and Research Service, Veterans Affairs Medical Center,

Iowa City, IA 52246, United States of America

(Z.Z., M.M.M., B.D.F., J.A.A., J.L.M/, M.L., W.N.S.).

Department of Internal Medicine, Roy G. and Lucille A. Carver College of Medicine, University of Iowa, Iowa City, IA 52242, United States of America

(Z.Z., H.T., J.L.M., W.N.S.)

Central Microscopy Research Facility Roy G. and Lucille A. Carver College of Medicine,

University of Iowa, Iowa City, IA 52242, United States of America

(T.O.M.)

**Supplemental Results.**

**Fig. S1. Structure of the MPPs (Metalloprotoporphyrins) used in these studies.**

**Fig S2. CoPP inhibited Taq DNA polymerase activity.**

As described in the main manuscript, we used TRAP assays with *Trapeze* to determine whether ZnPP, FePP, and SnPP inhibited telomerase activity similar to porphyrins (Figs 3B-D). Our procedure also tested for effects of the MPPs on *Taq* polymerase (*Taq),* a potential confounding interaction that has been reported for some other natural and synthetic porphyrins. In contrast to the minimal inhibition of *Taq* by ZnPP, FePP and SnPP, CoPP was a potent antagonist at concentrations of 5-10 µM (Figure S2). Note that *Taq* inhibition was also documented by the decreased activity of the internal control (IC), a *Taq* product, shown with SYBR fluorescence labelling, (Fig. S2, upper panels). These findings demonstrate that some MPPs can also have antagonistic effects on the activity of *Taq*, similar to some porphyrins and that these interactions are important to consider when comparing the effect of MPPs on telomerase activity. In spite of the selective effect of CoPP on Taq, we are currently assessing whether CoPP truly inhibits telomerase activity by direct α-^32^P-dGTP extension assay as we determined for ZnPP, FePP and SnPP (Fig. 3E, main manuscript). We recommend that MPPs be assayed by at least two direct extension assays until it is clear that a confounding interaction with *Taq* is not occurring.

**Fig S3. Labelling of cellular lysates with SnPP. Assessment of DNA and protein in ZnPP binding high molecular weight complexes.**

In contrast to ZnPP, another fluorescently active MPP, SnPP did not label a discernable cellular complex on agarose gels as compared to ZnPP (Fig. S3, upper panel). However, it should be noted that SnPP has weaker fluorescence intensity (3%) as compared to ZnPP (Lamola, 1981) (Tong et al., 2001) and detection of binding may be out of the resolution of this system. Even free SnPP was only imaged faintly on gels (Fig. S3). Further experiments to determine whether other fluorescently active MPPs, metal free protoporphyrins, common porphyrins, and derivatives are in progress.

We also used this experimental set up to conduct further experiments to look at protein and DNA composition of the separated complexes. Staining of complexes with SYBR green (Fig. S3 middle panel), which preferentially detects double strand (DS) DNA or RNA, showed that nearly all of the large DS nucleic acids stayed at the origin under non-denaturing conditions and were presumably too large to electrophorese into 0.8% agarose. SS DNA can also be labelled with SYBR green, but the fluorescence signal is 11 fold less than for DS DNA or RNA (Zipper et al., 2004) and may be out of the resolution of this system. When stained for protein with Coomassie blue, the extracts (Fig. S3 lower panel) showed significant protein throughout the electrophoretic field with multiple, slow mobility complexes; however, no prominent bands corresponded to the ZnPP labelled complexes. These data suggest that the complexes visualized by ZnPP are likely to be at low concentrations in crude cellular lysates. Further studies on the composition of complexes binding ZnPP were conducted with immunoblots, nuclease digestion, and TERT immunoprecipitation as described in the main manuscript.

**Fig. S4. Optimization of methods to transfer complexes from agarose gels to nitrocellulose sheets.**

In order to investigate ZnPP binding to high molecular weight complexes, it was necessary to optimize conditions for transfer of non-denatured complexes to nitrocellulose to allow immune-identification of components. We investigated several methods using electrophoretic and capillary diffusion conditions. In general, a capillary diffusion method using overnight times (14-16 hr) was optimal. In figure S4, we compared an electrophoretic transfer method with the capillary diffusion method shown in figure 4E of the main manuscript. The same cellular extracts were run on an identical gel, but electrophoretically transferred to nitrocellulose using routine 0.1% SDS Tris-glycine gel transfer buffer. Note that capillary diffusion proved to be much more effective than electrophoretic transfer and was adopted for all native immunoblots.

**Fig. S5. S phase synchrony of Huh7 cells with double thymidine block.**

As discussed in the main manuscript, it is clear that TERT preferentially associates with telomeric sites during cellular S phase. Consequently, to assess binding of ZnPP to telomeric sites and telomerase holoenzyme, we prepared S phase enriched Huh7 cell cultures using double thymidine blockade. The percentage of cells in S phase after release from thymidine block was assessed with Fluorescence Activated cell sorting (Fig. S5, upper panel). Cells were routinely used 2hr following release of thymidine blockade and at this time, 47.1 % of cells were in S phase; a more than doubling of enrichment. Enrichment of cells in S-phase was also confirmed with assay of Cyclin A2, a notable protein marker for S phase cells. These values are consistent with current expectations. (Chen et al., 2018)

References

Chen, G., Magis, A.T., Xu, K., Park, D., Yu, D.S., Owonikoko, T.K., Sica, G.L., Satola, S.W., Ramalingam, S.S., Curran, W.J., Doetsch, P.W., Deng, X., 2018. Targeting Mcl-1 enhances DNA replication stress sensitivity to cancer therapy. The Journal of clinical investigation 128, 500-516.

Lamola, A.A., 1981. Fluorescence methods in the diagnosis and management of diseases of tetrapyrrole metabolism. The Journal of investigative dermatology 77, 114-121.

Tong, A.j., Liu, L., Liu, L., Li, L.-d., Huie, C.W., 2001. Solid-substrate room-temperature phosphorescence study on zinc(II) and tin(IV) protoporphyrins and their interaction with DNA. Fresenius' Journal of Analytical Chemistry 370, 1023-1028.

Zipper, H., Brunner, H., Bernhagen, J., Vitzthum, F., 2004. Investigations on DNA intercalation and surface binding by SYBR Green I, its structure determination and methodological implications. Nucleic Acids Res 32, e103.

**Supplemental Figure Legends**

**Figure S2. CoPP inhibition of Taq Polymerase.**

Enzymatic extracts were prepared from semi-confluent Huh7 cells and aliquots were assayed in triplicate using TRAP assay. CoPP was added to RT-PCR reactions either before or after the RT telomere elongation step to test whether it inhibited *Taq* polymerase. Visualization of amplified telomeric products was on 12% denaturing gels using SYBR fluorescence labelling (upper panels). Quantification was with densitometry as described in Methods and plot shown (lower panel). Plotted points represent the mean +/- SD with n =3. (IC)= internal Taq polymerase control.

**Figure S3. Further characterization of MPP labelled complexes.**

Cellular lysates were prepared from log phase Huh7 cells and incubated 1hr at RT with various concentrations of SnPP or ZnPP for 1hr RT. Mixtures were electrophoresed on non-denaturing 0.8% agarose gels and visualized using broad band UV (upper panels), fluorescently with SYBR to label DNA and RNA (middle panels), or after staining with Coomassie Brilliant Blue to label protein lower panels).

**Figure S4 Comparison of capillary diffusion vs electrophoresis for transfer of 0.8% Agarose gel complexes to nitrocellulose (NC).**

Lysates from Huh7 cells were incubated for 1hr at RT with 10 µM ZnPP or vehicle control, then electrophoresed on 0.8% agarose gels and either diffusion-blotted overnight onto nitrocellulose (NC) using 1xTBS buffer (upper panels) or electrophoretically transferred to NC using a routine, widely employed, 0.1% SDS -Tris-glycine buffer system (lower panels). ZnPP complexes were visualized in gels by UV and on NC blots by red fluorescence. NC blots were then stained with specific anti-TERT or anti-dyskerin antibodies. The upper panels are reproduced from Fig. 4E in the main manuscript for direct comparison of the two transfer methods.

**Fig S5. Double thymidine block of Huh7 cells to synchronize in S phase.**

Huh7 cells were cultured using double thymidine blockade as described in Methods.

The percentage of cells in S phase was then determined at various times after release from blockade with Fluorescence Activated cell sorting methods (upper panel). Cellular lysates were also prepared at various times after blockade and the relative amounts of Cyclin A2 and TERT were determined on immunoblots (lower panels).
